# Multiple Pathways to the Same End: Mechanisms of Myonuclear Apoptosis in Sarcopenia of Aging

**DOI:** 10.1100/tsw.2010.27

**Published:** 2010-02-19

**Authors:** Emanuele Marzetti, Giuseppe Privitera, Vincenzo Simili, Stephanie E. Wohlgemuth, Lorenzo Aulisa, Marco Pahor, Christiaan Leeuwenburgh

**Affiliations:** ^1^ Department of Aging and Geriatric Research, Institute on Aging, Division of Biology of Aging, University of Florida, Gainesville, FL, USA; ^2^ Department of Orthopaedics and Traumatology, Catholic University of the Sacred Heart, Rome, Italy; ^3^ Department of Anesthesiology, Catholic University of the Sacred Heart, Rome, Italy

**Keywords:** aging, sarcopenia, myonuclear apoptosis, mitochondria, tumor necrosis factor-alpha, caspases, endonuclease G, apoptosis inducing factor

## Abstract

Sarcopenia, the age-related decline in muscle mass and function, represents a significant health issue due to the high prevalence of frailty and disability associated with this condition. Nevertheless, the cellular mechanisms responsible for the loss of muscle mass in old age are still largely unknown. An altered regulation of myocyte apoptosis has recently emerged as a possible contributor to the pathogenesis of sarcopenia. Studies in animal models have shown that the severity of skeletal muscle apoptosis increases over the course of aging and correlates with the degree of muscle mass and strength decline. Several apoptotic pathways are operative in aged muscles, with the mitochondria- and TNF-α-mediated pathways likely being the most relevant to sarcopenia. However, despite the growing number of studies on the subject, a definite mechanistic link between myocyte apoptosis and age-related muscle atrophy has not yet been established. Furthermore, the evidence on the role played by apoptosis in human sarcopenia is still sparse. Clearly, further research is required to better define the involvement of myocyte apoptosis in the pathogenesis of muscle loss at advanced age. This knowledge will likely help in the design of more effective therapeutic strategies to preserve muscle mass into old age, thus fostering independence of the elderly population and reducing the socioeconomic burden associated with sarcopenia.

## INTRODUCTION

The age-related loss of muscle mass and function, referred to as sarcopenia, is a highly prevalent condition among older persons, affecting 10–25% of the population under the age of 70 years, and over 40% of the individuals aged 80 years or older[1,2]. This age-dependent muscle loss has been reported even among healthy, physically active subjects and is associated with frailty, disability, poor quality of life, and increased mortality[3]. The loss of muscle mass in old age is often paralleled by a gain in fat mass, leading to the so-called "sarcopenic obesity" phenotype[4]. This syndrome is characterized by a vicious cycle, where muscle wasting lowers the resting metabolic rate and physical activity, with subsequent increase in adipose tissue deposition[4]. The accumulation of fat mass, in turn, promotes muscle loss via the secretion of catabolic cytokines (i.e., TNF-α) and insulin resistance[5].

The socioeconomic burden associated with sarcopenia has instigated intensive research on the factors responsible for this syndrome. Similar to many other age-related conditions, sarcopenia is characterized by a multifactorial etiology, in which neuronal[6] and hormonal[7] alterations, impaired satellite cell function[8], increased production of catabolic cytokines[9], poor nutrition[10], and decreased physical activity[7], cooperate in the pathogenesis of the decline in muscle mass. However, the cellular mechanisms ultimately leading to muscle loss are still largely unknown.

Over the past few years, evidence has accumulated suggesting that an accelerated elimination of myonuclei via an apoptosis-like process might play a significant role in the loss of muscle mass and function with age[11,12,13,14,15,16,17,18,19,20]. A key feature of apoptosis in skeletal muscle is linked to the multinucleated nature of myofibers, where single nuclei (and their portion of cytoplasm) can be eliminated without the dismantling of the whole fiber. The relevance of this unique process (myonuclear apoptosis) to sarcopenia as well as the signaling transduction pathways activated during aging will be discussed in this brief review.

## SIGNALING PATHWAYS OF APOPTOTIC CELL DEATH

Apoptosis, a process of programmed cell death, is an evolutionarily conserved, tightly regulated, systematic set of events resulting in cellular self-destruction without inflammation or damage to the surrounding tissue[21]. Apoptosis plays a fundamental role in the development and maintenance of tissue homeostasis. Proteolytic enzymes, known as caspases, perform the dismantling of the cell and are normally present as inactive zymogens (procaspases)[22]. Upon appropriate stimuli, initiator caspases (i.e., caspase-8, caspase-9, caspase-12) are activated, leading to the activation of effector caspases (i.e., caspase-3, caspase-6, caspase-7) responsible for the cellular degradation and DNA fragmentation via a caspase-activated DNase (CAD)[23]. Activation of caspases can occur through extrinsic and intrinsic pathways (Fig. 1)[22]. The extrinsic apoptotic signaling is initiated by death receptors located on the cell surface, such as the tumor necrosis factor receptor (TNF-R) and the Fas receptor[24]. Activation of these receptors by binding of death-stimulating ligands results in the recruitment of adaptor proteins, such as Fas-associated death domain (FADD), TNF-receptor-associated death domain (TRADD), and TNF-receptor-associated factors (TRAFs), forming the death-inducing signaling complex (DISC), which engages and activates procaspase-8. Active caspase-8 subsequently activates caspase-3[24]. Intrinsic pathways of caspase activation include those triggered by the endoplasmic reticulum (ER) and the mitochondrion[22]. Under stress conditions, such as calcium dyshomeostasis, the ER-specific procaspase-12 can be activated by m-calpain, leading to caspase-3 activation[25]. However, the mitochondria-mediated intrinsic signaling pathway of apoptosis is currently considered the most relevant means of programmed cell death and has been the subject of intense scientific focus[26].

Notably, mitochondria can induce apoptosis via both caspase-mediated and caspase-independent pathways[26]. The former is initiated by the release of cytochrome *c* into the cytoplasm, where it complexes with apoptotic protease-activating factor-1 (Apaf-1), dATP, and procaspase-9, forming the apoptosome[26]. Within this complex, procaspase-9 becomes activated, subsequently engaging caspase-3[26]. In addition, mitochondria can release the second mitochondria-derived activator of caspases/direct inhibitor of apoptosis-binding protein with low pI (Smac/DIABLO) and heat requirement A2 protein (Omi/HtrA2), both of which block the activity of the inhibitor of apoptosis proteins (IAPs)[27]. Hence, inhibition of caspase activity is removed and caspase-mediated proteolysis proceeds.

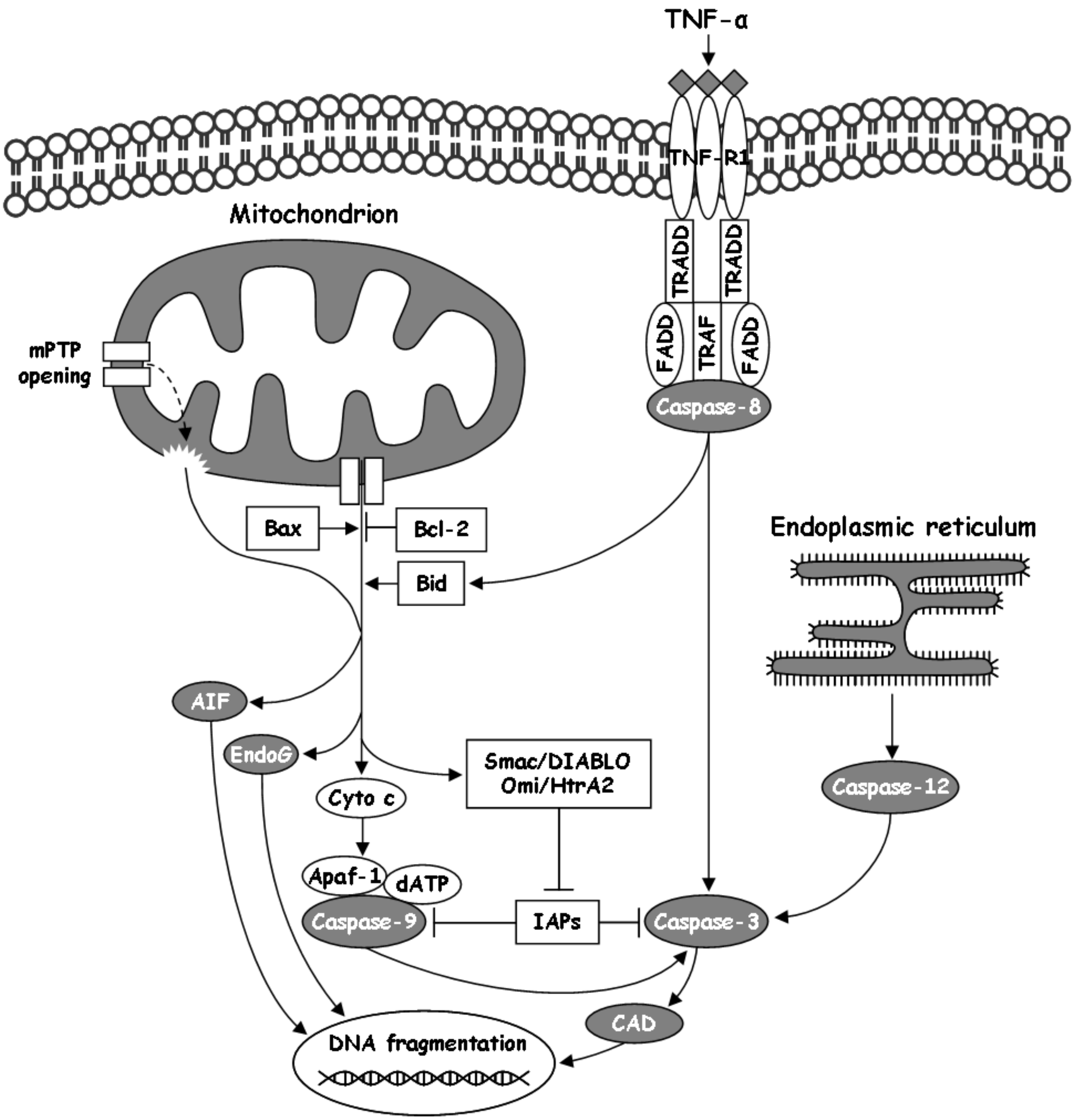

**FIGURE 1.** Schematic overview of signaling pathways of myonuclear apoptosis activated at advanced age.

Aside from their role in cytochrome *c*–mediated induction of cell death, mitochondria also participate in a pathway of apoptosis that does not involve caspase activation. Endonuclease G (EndoG) and apoptosis inducing factor (AIF) are two mediators that, upon release from mitochondria, translocate to the nucleus and perform DNA fragmentation independent of caspases[27,28]. Specifically, AIF binds to DNA to induce chromatin condensation[28]. AIF can also interact with cyclophilin A, forming a DNase responsible for large-scale DNA fragmentation[29]. EndoG, a mitochondrion-specific nuclease, is thought to participate directly in oligonucleosomal DNA fragmentation[30].

Upstream of the release of mitochondria-specific apoptotic mediators is the process of mitochondrial outer membrane permeabilization (MOMP)[31], whose intimate mechanisms are currently under intense investigation. The integrity of the outer mitochondrial membrane (OMM) is regulated by the Bcl-2 family of proteins that includes pro- (e.g., Bax, Bak, Bad, Bim, Bid, Puma, Noxa) and antiapoptotic (e.g., Bcl-2, Bcl-$X_L$, Bcl-w, Mcl-1, A1) members[32]. The balance between these mediators (e.g., Bax-to-Bcl2 ratio) is considered a fundamental control point for the cell fate by regulating OMM stability. Under normal conditions, Bcl-2 and Bcl-$X_L$ block Bax and/or Bak[33]. In apoptotic conditions, Bax translocates from the cytosol to the mitochondria, oligomerizes, and inserts into the OMM in concentrated foci[34]. This process results in the formation of a pore through which apoptotic factors stored in the intermembrane compartment are released[34]. Similarly, Bak, which is constitutively anchored to the OMM, can form homo-oligomeric complexes upon apoptotic stimulation[34]. Bax/Bak hetero-oligomers have also been described[35]. The proapoptotic factors Bid and Bim participate to Bax and Bak activation, through neutralizing Bcl-2 and Bcl-$X_L$ activity[36]. In addition, Bid allows cross-talk between intrinsic and extrinsic apoptotic pathways, after truncation by caspase-8[37].

MOMP is also thought to occur via opening of the mitochondrial permeability transition pore (mPTP)[38]. The exact nature of the mPTP is still unclear. However, three putative components have been suggested: a voltage-dependent anion channel (VDAC) located in the OMM, the adenine nucleotide translocase (ANT) in the inner mitochondrial membrane (IMM), and cyclophilin D (CyPD) in the matrix[39]. Upon certain stimuli, such as oxidative stress or calcium overload, ANT interacts with VDAC, generating an unselective pore spanning the IMM and OMM[40]. This interaction is stabilized by CyPD. Opening of the mPTP causes a sudden increase in membrane permeability to solutes with molecular weight up to 1,500 Da. This results in dissipation of membrane potential, mitochondrial swelling, and rupture of the OMM, with subsequent release of mitochondrial apoptotic mediators[38]. Interestingly, cross-talk between the mPTP and Bcl-2 proteins has been described, with Bid and Bax promoting mPTP opening[41,42], while the antiapoptotic Bcl-2 and Bcl-$X_L$ possess an inhibitory effect[43].

## THE INVOLVEMENT OF APOPTOSIS IN THE PATHOGENESIS OF SARCOPENIA

A wealth of experimental animal data indicates that the apoptotic program is activated in the aged skeletal muscle, likely contributing to the pathogenesis of sarcopenia (Fig. 1)[44].

Given the central role played by mitochondria in the induction and regulation of programmed cell death, intensive investigation has focused on mitochondria-driven myonuclear apoptosis[45].

Alteration in the expression of Bcl-2 family proteins has been reported in skeletal muscles of aged experimental animals[18,46,47,48,49]. Specifically, a proapoptotic shift in the expression pattern of Bcl-2 proteins (i.e., increased Bax and decreased Bcl-2 levels) has been observed in muscles of aged rodents[47,48,49]. However, other studies did not detect such an adaptation[18,46]. Strikingly, lower levels of Bax and Bcl-2 mRNA were found in the plantaris muscle of old and senescent rats, resulting in a reduced, rather than increased, Bax-to-Bcl-2 ratio in very old animals compared to younger controls[50]. Interestingly, the age-related pattern of Bcl-2 and Bax expression may be muscle-type specific[51]. In fact, Bax content was found to be increased in the fast-twitch extensor digitorum longus (EDL) of old rats, whereas no changes were observed in the slow-twitch soleus. In contrast, both muscles displayed an increased Bcl-2 expression at advanced age. The elevation of Bcl-2 detected in aged muscles may be interpreted as a compensatory attempt to limit myonuclear loss. Alternatively, the increased serine-phosphorylation and subsequent inactivation of Bcl-2 recently reported in old mice[52] might abolish the antiapoptotic activity of this molecule despite elevated expression[53].

Besides alterations in the Bcl-2 rheostat, increased susceptibility towards opening of the mPTP has also been demonstrated in aged skeletal muscles[54,55,56]. Interestingly, elevated mitochondrial levels of CyPD relative to ANT and VDAC were detected in the gastrocnemius muscle of very old rats[18]. This finding underlines the major role postulated for CyPD in the formation of the mPTP, and suggests a higher susceptibility to mPTP opening at an older age. Indeed, it was demonstrated that mPTP formation and apoptotic cell death could still occur in the absence of either ANT[57] or VDAC[58]. In contrast, overexpression of CyPD induced mitochondrial swelling and spontaneous cardiac apoptosis, whereas mice lacking cardiac CyPD were protected from ischemia/reperfusion-induced cell death[59].

Once MOMP has occurred, the release of apoptogenic factors stored in the mitochondrial intermembrane space ensues, initiating the series of events that culminate in cell death[31]. Elevated cytosolic levels of cytochrome *c* have been detected in the skeletal muscle of old rats[46]. Accordingly, levels of Apaf-1[13,46,60], active caspase-9 content[52,54,60], and caspase-9 proteolytic activity[46] were also found to be higher in aged muscles compared to young controls. However, other studies did not observe increases in cytosolic cytochrome *c* and/or caspase-9 expression in skeletal muscles of old rats[12,13,18,49]. Surprisingly, it was observed that gene expression levels of Apaf-1 were even reduced in the plantaris of old and senescent rats relative to younger controls[50].

These observations have cast doubts about the involvement of mitochondrial caspase-dependent apoptosis in the pathogenesis of sarcopenia. In contrast, several studies have shown that caspase-independent apoptotic pathways are activated in old age[12,18,50,61]. It was found that translocation of mitochondrial EndoG to the nucleus was increased in the soleus muscle of old rats. In addition, an age-related increase in both cytosolic and nuclear levels of AIF and EndoG was observed in the rat gastrocnemius muscle[18]. Moreover, AIF gene expression progressively increased during aging in the rat plantaris muscle, and correlated with the progression of sarcopenia[50]. Collectively, these findings support a role for the mitochondrial caspase-independent pathway of apoptosis in the pathophysiology of sarcopenia. Indeed, this apoptotic pathway might be particularly relevant in sarcopenia, since it may allow for the elimination of superfluous/damaged nuclei without the demolition of the entire muscle fiber by caspases[62].

Besides mitochondria-driven apoptosis, other pathways of myonuclear apoptosis have been found to be operative at advanced age. In particular, the death receptor–mediated pathway, triggered by TNF-α, is thought to play a significant role in age-related muscle loss[14,17,19,20], owing to its ability to promote muscle protein wasting[63,64] and myonuclear apoptosis[14,17,19,20,65]. Interestingly, the transduction of TNF-α signaling during aging may be fiber-type specific[14]. In fact, TNF-α–mediated apoptosis was enhanced at old age in the rat superficial vastus lateralis muscle (predominantly comprised of type II fibers), whereas increased signaling through the NF-κB pathway was observed in the soleus muscle, characterized by type I fiber dominance. Notably, NF-κB may promote the expression of antiapoptotic genes such as cIAP-1, cIAP-2, and FLICE-like inhibitory protein long form ($FLIP_L$)[66,67]. This phenomenon could at least partly explain the lower susceptibility to age-related atrophy displayed by slow-twitch muscles compared to those with fast-twitch properties[68]. In support of these findings, Marzetti et al.[19] recently detected an age-related increase in myocyte apoptosis in the rat extensor digitorum longus (EDL, type II) muscle, whereas the extent of DNA fragmentation was unaffected by age in the soleus. Furthermore, the death receptor pathway of apoptosis was activated in the EDL, but not in the soleus of aged rats. Interestingly, 4-week treadmill exercise training reversed the age-related elevation of DNA fragmentation and TNF-α apoptotic signaling in the EDL muscle. Importantly, mitigation in the extent of apoptosis was accompanied by improved exercise tolerance and grip strength, suggesting a strong association between the severity of myocyte apoptosis and physical performance. In another very recent study, the age-dependent increase in TNF-α apoptotic signaling was paralleled by decreased expression of the antiapoptotic interleukin 15 (IL-15) and its specific receptor subunit α (IL-15Rα) in the rat gastrocnemius muscle[20]. In contrast, lifelong calorie restriction (CR), an intervention known to attenuate apoptosis in the skeletal muscle of old rodents[69], preserved IL-15 signaling and muscle mass into very old age, while preventing the activation of the death receptor–mediated apoptosis[20]. These findings suggest that the loss of the anabolic drive by IL-15 may unbridle the proapoptotic actions of TNF-α in skeletal muscle, thus contributing to the development of sarcopenia and loss of physical function in old age.

Similar to the TNF-α/IL-15 axis dysfunction, an altered balance between insulin-like growth factor-1 (IGF-1) and TNF-α may also contribute to the proapoptotic environment taking place in the aged muscle. IGF-1, either systemic or muscle derived, is a potent stimulator of muscle development, hypertrophy, and maintenance[70]. Importantly, IGF-1 is also able to counteract the proapoptotic actions of TNF-α in skeletal muscle. Indeed, incubation of C2 myoblasts with IGF-I decreases the incidence of TNF-α–induced apoptosis[71]. Levels of IGF-1 are reduced with advanced age, as a consequence of impaired growth hormone (GH) production[72]. In addition, muscle production of mechano growth factor (MGF), a local splice variant of IGF-1, is decreased in old rats in response to mechanical overload[73]. Furthermore, both the density and affinity of the IGF type 1 receptor are reduced in the aged muscle[74]. Downstream of IGF-1 is the protein kinase B (PKB), also known as Akt. Akt plays a central role in muscle anabolism by stimulating glucose uptake, glycogen synthesis, and protein synthesis[75]. Akt also inhibits myonuclear apoptosis and muscle protein degradation by inactivating Bad[76] and FoxO transcription factors[77], respectively. Insulin-stimulated Akt activation is significantly decreased in advanced age in the rat skeletal muscle[78], as a result of TNF-α–induced insulin resistance[79]. Interestingly, Wu et al.[80] recently reported an age-related decrease in Akt kinase activity in the rat skeletal muscle, which was associated with elevated levels of Akt S-nitrosylation. Noteworthy, amelioration of Akt activity following acetaminophen administration was accompanied by attenuation of myonuclear apoptosis and increases in myocyte cross-sectional area and contractile protein expression.

Other apoptotic pathways that may be operative in the aged muscle include those initiated by caspase-2 and -12; however, their contribution to sarcopenia has yet to be established. Braga et al.[52] recently found an increased caspase-2 expression in the skeletal muscle of old mice. This caspase is thought to cooperate with MOMP during stress-induced apoptosis[81]. However, it is still unclear whether caspase-2 is an initiator or an effector caspase and how it is activated.

Finally, recent evidence suggests that the ER-induced apoptosis may participate to age-related muscle atrophy. In fact, increased caspase-12 expression was detected in the skeletal muscle of old rats[13,60,82], suggesting a role for the ER stress response in sarcopenia.

## THE INVOLVEMENT OF MYOCYTE APOPTOSIS IN HUMAN SARCOPENIA

Although studies in animal models appear to support a key role for apoptosis in age-related muscle atrophy, evidence in humans is still lacking. To date, only three reports have been published examining the occurrence of myocyte apoptosis in older persons[11,16,83]. However, none of these studies has examined either specific biochemical pathways of apoptosis or functional implications. Interestingly, Whitman et al.[16] found that the increase in apoptotic myonuclei in older persons was not accompanied by changes in caspase-3 and -7 activity. This may indicate that the caspase-independent pathway of myonuclear apoptosis might be selectively activated during aging, as reported by Marzetti et al.[18], in aged rats. In support of this hypothesis, Park et al.[84] recently found increased expression of AIF in the semi-tendinosus muscle of middle-aged men relative to younger controls, whereas no age-related changes in caspase-3 levels were detected. Clearly, further research is required in order to understand the signaling transduction pathways of myonuclear apoptosis in older human subjects, as well as their contribution to sarcopenia and physical function decline.

## CONCLUSIONS

Growing evidence indicates that an age-related deregulation of apoptosis may contribute to the onset and progression of sarcopenia. However, several critical issues are still unsolved. First, a definite mechanistic link between myonuclear apoptosis and muscle loss has yet to be established. Furthermore, the relative contribution of the various apoptotic pathways to sarcopenia is still unresolved. Likewise, it is unclear whether specific signaling transduction pathways are selectively activated during the progression of muscle wasting. Finally, the role of apoptosis in human sarcopenia warrants further investigation. In particular, a deeper understanding is necessary regarding the relationship between the severity of myonuclear apoptosis, muscle atrophy, and physical function decline in older persons. By answering these fundamental research questions, novel biomarkers of sarcopenia will be provided that may be utilized for the development of new therapeutic remedies for sarcopenia and functional decline in old age.

## ACKNOWLEDGMENTS

This research was supported by grants to CL (NIA R01-AG17994 and AG21042), and by the University of Florida Institute on Aging and Claude D. Pepper Older Americans Independence Center (1 P30AG028740).

## References

1. Baumgartner, R.N., Koehler, K.M., Gallagher, D., Romero, L., Heymsfield, S.B., Ross, R.R., Garry, P.J., and Lindeman, R.D. (1998) Epidemiology of sarcopenia among the elderly in New Mexico. *Am. J. Epidemiol*. **147**, 755–763.
2. Iannuzzi-Sucich, M., Prestwood, K.M., and Kenny, A.M. (2002) Prevalence of sarcopenia and predictors of skeletal muscle mass in healthy, older men and women. *J. Gerontol. A Biol. Sci. Med. Sci.* **57**, M772–M777.
3. Marzetti, E. and Leeuwenburgh, C. (2006) Skeletal muscle apoptosis, sarcopenia and frailty at old age. *Exp. Gerontol*. **41**, 1234–1238.
4. Roubenoff, R. (2004) Sarcopenic obesity: the confluence of two epidemics. *Obes. Res.* **12**, 887–888.
5. Bastard, J.P., Maachi, M., Lagathu, C., Kim, M.J., Caron, M., Vidal, H., Capeau, J., and Feve, B. (2006) Recent advances in the relationship between obesity, inflammation, and insulin resistance. *Eur. Cytokine Netw*. **17**, 4–12.
6. Vandervoort, A.A. (2002) Aging of the human neuromuscular system. *Muscle Nerve* **25**, 17–25.
7. Szulc, P., Duboeuf, F., Marchand, F., and Delmas, P.D. (2004) Hormonal and lifestyle determinants of appendicular skeletal muscle mass in men: the MINOS study. *Am. J. Clin. Nutr.* **80**, 496–503.
8. Lees, S.J., Rathbone, C.R., and Booth, F.W. (2006) Age-associated decrease in muscle precursor cell differentiation. *Am. J. Physiol. Cell Physiol*. **290**, C609–C615.
9. Visser, M., Pahor, M., Taaffe, D.R., Goodpaster, B.H., Simonsick, E.M., Newman, A.B., Nevitt, M., and Harris, T.B. (2002) Relationship of interleukin-6 and tumor necrosis factor-alpha with muscle mass and muscle strength in elderly men and women: the Health ABC Study. *J. Gerontol. A Biol. Sci. Med. Sci*. **57**, M326–M332.
10. Dreyer, H.C. and Volpi, E. (2005) Role of protein and amino acids in the pathophysiology and treatment of sarcopenia. *J. Am. Coll. Nutr*. **24**, 140S–145S.
11. Strasser, H., Tiefenthaler, M., Steinlechner, M., Bartsch, G., and Konwalinka, G. (1999) Urinary incontinence in the elderly and age-dependent apoptosis of rhabdosphincter cells. *Lancet* **354**, 918–919.
12. Dirks, A. and Leeuwenburgh, C. (2002) Apoptosis in skeletal muscle with aging. *Am. J. Physiol Regul. Integr. Comp. Physiol.* **282**, R519–R527.
13. Dirks, A.J. and Leeuwenburgh, C. (2004) Aging and lifelong calorie restriction result in adaptations of skeletal muscle apoptosis repressor, apoptosis-inducing factor, X-linked inhibitor of apoptosis, caspase-3, and caspase-12. *Free Radic. Biol. Med*. **36**, 27–39.
14. Phillips, T. and Leeuwenburgh, C. (2005) Muscle fiber specific apoptosis and TNF-alpha signaling in sarcopenia are attenuated by life-long calorie restriction. *FASEB J.* **19**, 668–670.
15. Siu, P.M., Pistilli, E.E., Butler, D.C., and Alway, S.E. (2005) Aging influences cellular and molecular responses of apoptosis to skeletal muscle unloading. *Am. J. Physiol. Cell Physiol.* **288**, C338–C349.
16. Whitman, S.A., Wacker, M.J., Richmond, S.R., and Godard, M.P. (2005) Contributions of the ubiquitin-proteasome pathway and apoptosis to human skeletal muscle wasting with age. *Pflugers Arch*. **450**, 437–446.
17. Pistilli, E.E., Jackson, J.R., and Alway, S.E. (2006) Death receptor-associated pro-apoptotic signaling in aged skeletal muscle. *Apoptosis* **11**, 2115–2126.
18. Marzetti, E., Wohlgemuth, S.E., Lees, H.A., Chung, H.Y., Giovannini, S., and Leeuwenburgh, C. (2008) Age-related activation of mitochondrial caspase-independent apoptotic signaling in rat gastrocnemius muscle. *Mech. Ageing Dev*. **129**, 542–549.
19. Marzetti, E., Groban, L., Wohlgemuth, S.E., Lees, H.A., Lin, M., Jobe, H., Giovannini, S., Leeuwenburgh, C., and Carter, C.S. (2008) Effects of short-term GH supplementation and treadmill exercise training on physical performance and skeletal muscle apoptosis in old rats. *Am. J. Physiol. Regul. Integr. Comp. Physiol*. **294**, R558–R567.
20. Marzetti, E., Carter, C.S., Wohlgemuth, S.E., Lees, H.A., Giovannini, S., Anderson, B., Quinn, L.S., and Leeuwenburgh, C. (2009) Changes in IL-15 expression and death-receptor apoptotic signaling in rat gastrocnemius muscle with aging and life-long calorie restriction. *Mech. Ageing Dev*. **130**, 272–280.
21. Kerr, J.F., Wyllie, A.H., and Currie, A.R. (1972) Apoptosis: a basic biological phenomenon with wide-ranging implications in tissue kinetics. *Br. J. Cancer* **26**, 239–257.

22. Danial, N.N. and Korsmeyer, S.J. (2004) Cell death: critical control points. *Cell* **116**, 205–219.
23. Hengartner, M.O. (2000) The biochemistry of apoptosis. *Nature* **407,** 770–776.
24. Wang, Z.B., Liu, Y.Q., and Cui, Y.F. (2005) Pathways to caspase activation. *Cell Biol. Int.* **29**, 489–496.
25. Nakagawa, T. and Yuan, J. (2000) Cross-talk between two cysteine protease families. Activation of caspase-12 by calpain in apoptosis. *J. Cell Biol*. **150,** 887–894.
26. Jeong, S.Y. and Seol, D.W. (2008) The role of mitochondria in apoptosis. *BMB Rep.* **41**, 11–22.
27. van Loo, G., Saelens, X., van Gurp, M., MacFarlane, M., Martin, S.J., and Vandenabeele, P. (2002) The role of mitochondrial factors in apoptosis: a Russian roulette with more than one bullet. *Cell Death Differ*. **9**, 1031–1042.
28. Daugas, E., Nochy, D., Ravagnan, L., Loeffler, M., Susin, S.A., Zamzami, N., and Kroemer, G. (2000) Apoptosis-inducing factor (AIF): a ubiquitous mitochondrial oxidoreductase involved in apoptosis. *FEBS Lett*. **476**, 118–123.
29. Cande, C., Vahsen, N., Kouranti, I., Schmitt, E., Daugas, E., Spahr, C., Luban, J., Kroemer, R.T., Giordanetto, F., Garrido, C., Penninger, J.M., and Kroemer, G. (2004) AIF and cyclophilin A cooperate in apoptosis-associated chromatinolysis. *Oncogene* **23**, 1514–1521.
30. Huang, K.J., Ku, C.C., and Lehman, I.R. (2006) Endonuclease G: a role for the enzyme in recombination and cellular proliferation. *Proc. Natl. Acad. Sci. U. S. A.* **103**, 8995–9000.
31. Chipuk, J.E., Bouchier-Hayes, L., and Green, D.R. (2006) Mitochondrial outer membrane permeabilization during apoptosis: the innocent bystander scenario. *Cell* Death Differ. **13**, 1396–1402.
32. Chao, D.T. and Korsmeyer, S.J. (1998) BCL-2 family: regulators of cell death. *Annu. Rev. Immunol.* **16,** 395–419.
33. Antonsson, B., Conti, F., Ciavatta, A., Montessuit, S., Lewis, S., Martinou, I., Bernasconi, L., Bernard, A., Mermod, J.J., Mazzei, G., Maundrell, K., Gambale, F., Sadoul, R., and Martinou, J.C. (1997) Inhibition of Bax channel-forming activity by Bcl-2. *Science* **277**, 370–372.
34. van Delft, M.F. and Huang, D.C. (2006) How the Bcl-2 family of proteins interact to regulate apoptosis. *Cell Res*. **16**, 203–213.
35. Zhou, L. and Chang, D.C. (2008) Dynamics and structure of the Bax-Bak complex responsible for releasing mitochondrial proteins during apoptosis. *J. Cell Sci*. **121**, 2186–2196.
36. Chipuk, J.E. and Green, D.R. (2008) How do BCL-2 proteins induce mitochondrial outer membrane permeabilization? *Trends Cell Biol*. **18**, 157–164.
37. Li, H., Zhu, H., Xu, C.J., and Yuan, J. (1998) Cleavage of BID by caspase 8 mediates the mitochondrial damage in the Fas pathway of apoptosis. *Cell* **94**, 491–501.
38. Kroemer, G., Galluzzi, L., and Brenner, C. (2007) Mitochondrial membrane permeabilization in cell death. *Physiol. Rev*. **87**, 99–163.
39. Crompton, M., Virji, S., and Ward, J.M. (1998) Cyclophilin-D binds strongly to complexes of the voltage-dependent anion channel and the adenine nucleotide translocase to form the permeability transition pore. *Eur. J. Biochem*. **258,** 729–735.
40. Tsujimoto, Y. and Shimizu, S. (2007) Role of the mitochondrial membrane permeability transition in cell death. *Apoptosis* **12**, 835–840.
41. Marzo, I., Brenner, C., Zamzami, N., Jurgensmeier, J.M., Susin, S.A., Vieira, H.L., Prevost, M.C., Xie, Z., Matsuyama, S., Reed, J.C., and Kroemer, G. (1998) Bax and adenine nucleotide translocator cooperate in the mitochondrial control of apoptosis. *Science* **281**, 2027–2031.
42. Zamzami, N., El Hamel, C., Maisse, C., Brenner, C., Munoz-Pinedo, C., Belzacq, A.S., Costantini, P., Vieira, H., Loeffler, M., Molle, G., and Kroemer, G. (2000) Bid acts on the permeability transition pore complex to induce apoptosis. *Oncogene* **19**, 6342–6350.
43. Zamzami, N., Brenner, C., Marzo, I., Susin, S.A., and Kroemer, G. (1998) Subcellular and submitochondrial mode of action of Bcl-2-like oncoproteins. *Oncogene* **16,** 2265–2282.
44. Marzetti, E., Lawler, J.M., Hiona, A., Manini, T., Seo, A.Y., and Leeuwenburgh, C. (2008) Modulation of age-induced apoptotic signaling and cellular remodeling by exercise and calorie restriction in skeletal muscle. *Free Radic. Biol. Med*. **44**, 160–168.
45. Marzetti, E., Hwang, J.C., Lees, H.A., Wohlgemuth, S.E., Dupont-Versteegden, E.E., Carter, C.S., Bernabei, R., and Leeuwenburgh, C. (2009) Mitochondrial death effectors: relevance to sarcopenia and disuse muscle atrophy. *Biochim. Biophys. Acta* [Epub ahead of print]
46. Siu, P.M., Pistilli, E.E., and Alway, S.E. (2005) Apoptotic responses to hindlimb suspension in gastrocnemius muscles from young adult and aged rats. *Am. J. Physiol. Regul. Integr. Comp. Physiol.* **289**, R1015–R1026.
47. Alway, S.E., Degens, H., Krishnamurthy, G., and Smith, C.A. (2002) Potential role for Id myogenic repressors in apoptosis and attenuation of hypertrophy in muscles of aged rats. *Am. J. Physiol. Cell Physiol*. **283**, C66–C76.
48. Song, W., Kwak, H.B., and Lawler, J.M. (2006) Exercise training attenuates age-induced changes in apoptotic signaling in rat skeletal muscle. *Antioxid. Redox Signal*. **8**, 517–528.
49. Pistilli, E.E., Siu, P.M., and Alway, S.E. (2006) Molecular regulation of apoptosis in fast plantaris muscles of aged rats. *J. Gerontol. A Biol. Sci. Med. Sci*. **61,** 245–255.
50. Baker, D.J. and Hepple, R.T. (2006) Elevated caspase and AIF gene expression correlate with progression of sarcopenia during aging in male F344BN rats. *Exp. Gerontol.* **41**, 1149–1156.
51. Rice, K.M. and Blough, E.R. (2006) Sarcopenia-related apoptosis is regulated differently in fast- and slow-twitch muscles of the aging F344/N x BN rat model. *Mech. Ageing Dev.* **127**, 670–679.

52. Braga, M., Sinha Hikim, A.P., Datta, S., Ferrini, M.G., Brown, D., Kovacheva, E.L., Gonzalez-Cadavid, N.F., and Sinha-Hikim, I. (2008) Involvement of oxidative stress and caspase 2-mediated intrinsic pathway signaling in age-related increase in muscle cell apoptosis in mice. *Apoptosis* **13**, 822–832.
53. Rajah, R., Lee, K.W., and Cohen, P. (2002) Insulin-like growth factor binding protein-3 mediates tumor necrosis factor-alpha-induced apoptosis: role of Bcl-2 phosphorylation. *Cell Growth Differ*. **13**, 163–171.
54. Tamilselvan, J., Jayaraman, G., Sivarajan, K., and Panneerselvam, C. (2007) Age-dependent upregulation of p53 and cytochrome c release and susceptibility to apoptosis in skeletal muscle fiber of aged rats: role of carnitine and lipoic acid. *Free Radic. Biol. Med.* **43,** 1656–1669.
55. Chabi, B., Ljubicic, V., Menzies, K.J., Huang, J.H., Saleem, A., and Hood, D.A. (2008) Mitochondrial function and apoptotic susceptibility in aging skeletal muscle. *Aging Cell* **7**, 2–12.
56. Seo, A.Y., Xu, J., Servais, S., Hofer, T., Marzetti, E., Wohlgemuth, S.E., Knutson, M.D., Chung, H.Y., and Leeuwenburgh, C. (2008) Mitochondrial iron accumulation with age and functional consequences. *Aging Cell* **7,** 706–716.
57. Kokoszka, J.E., Waymire, K.G., Levy, S.E., Sligh, J.E., Cai, J., Jones, D.P., MacGregor, G.R., and Wallace, D.C. (2004) The ADP/ATP translocator is not essential for the mitochondrial permeability transition pore. *Nature* **427**, 461–465.
58. Baines, C.P., Kaiser, R.A., Sheiko, T., Craigen, W.J., and Molkentin, J.D. (2007) Voltage-dependent anion channels are dispensable for mitochondrial-dependent cell death. *Nat. Cell Biol*. **9**, 550–555.
59. Baines, C.P., Kaiser, R.A., Purcell, N.H., Blair, N.S., Osinska, H., Hambleton, M.A., Brunskill, E.W., Sayen, M.R., Gottlieb, R.A., Dorn, G.W., Robbins, J., and Molkentin, J.D. (2005) Loss of cyclophilin D reveals a critical role for mitochondrial permeability transition in cell death. *Nature* **434**, 658–662.
60. Chung, L. and Ng, Y.C. (2006) Age-related alterations in expression of apoptosis regulatory proteins and heat shock proteins in rat skeletal muscle. *Biochim. Biophys. Acta* **1762**, 103–109.
61. Leeuwenburgh, C., Gurley, C.M., Strotman, B.A., and Dupont-Versteegden, E.E. (2005) Age-related differences in apoptosis with disuse atrophy in soleus muscle. *Am. J. Physiol. Regul. Integr. Comp. Physiol.* **288** R1288–R1296.
62. Dupont-Versteegden, E.E. (2005) Apoptosis in muscle atrophy: relevance to sarcopenia. *Exp. Gerontol.* **40**, 473–481.
63. Garcia-Martinez, C., Agell, N., Llovera, M., Lopez-Soriano, F.J., and Argiles, J.M. (1993) Tumour necrosis factor-alpha increases the ubiquitinization of rat skeletal muscle proteins. *FEBS Lett.* **323**, 211–214.
64. Llovera, M., Lopez-Soriano, F.J., and Argiles, J.M. (1993) Effects of tumor necrosis factor-alpha on muscle-protein turnover in female Wistar rats. *J. Natl. Cancer Inst*. **85,** 1334–1339.
65. Carbo, N., Busquets, S., van Royen, M., Alvarez, B., Lopez-Soriano, F.J., and Argiles, J.M. (2002) TNF-alpha is involved in activating DNA fragmentation in skeletal muscle. *Br. J. Cancer* **86**, 1012–1016.
66. Wang, C.Y., Mayo, M.W., Korneluk, R.G., Goeddel, D.V., and Baldwin, A.S., Jr. (1998) NF-kappaB antiapoptosis: induction of TRAF1 and TRAF2 and c-IAP1 and c-IAP2 to suppress caspase-8 activation. *Science* **281**, 1680–1683.
67. Micheau, O., Lens, S., Gaide, O., Alevizopoulos, K., and Tschopp, J. (2001) NF-kappaB signals induce the expression of c-FLIP. *Mol. Cell. Biol*. **21**, 5299–5305.
68. Lexell, J. (1995) Human aging, muscle mass, and fiber type composition. *J. Gerontol. A Biol. Sci. Med. Sci.* **50(Spec. No.)**, 11–16.
69. Marzetti, E., Lees, H.L., Wohlgemuth, S.E., and Leeuwenburgh, C. (2009) Sarcopenia of aging: underlying cellular mechanisms and protection by calorie restriction. *BioFactors* **35**, 28–35.
70. Clemmons, D.R. (2009) Role of IGF-I in skeletal muscle mass maintenance. *Trends Endocrinol. Metab.* **20**, 349–356.
71. Saini, A., Al-Shanti, N., Faulkner, S.H., and Stewart, C.E. (2008) Pro- and anti-apoptotic roles for IGF-I in TNF-alpha-induced apoptosis: a MAP kinase mediated mechanism. *Growth Factors* **26**, 239–253.
72. Martin, F.C., Yeo, A.L., and Sonksen, P.H. (1997) Growth hormone secretion in the elderly: ageing and the somatopause. *Baillieres Clin. Endocrinol. Metab*. **11**, 223–250.
73. Owino, V., Yang, S.Y., and Goldspink, G. (2001) Age-related loss of skeletal muscle function and the inability to express the autocrine form of insulin-like growth factor-1 (MGF) in response to mechanical overload. *FEBS Lett*. **505**, 259–263.
74. Martineau, L.C., Chadan, S.G., and Parkhouse, W.S. (1999) Age-associated alterations in cardiac and skeletal muscle glucose transporters, insulin and IGF-1 receptors, and PI3-kinase protein contents in the C57BL/6 mouse. *Mech. Ageing Dev*. **106**, 217–232.
75. Frost, R.A. and Lang, C.H. (2007) Protein kinase B/Akt: a nexus of growth factor and cytokine signaling in determining muscle mass. *J. Appl. Physiol*. **103**, 378–387.
76. Gao, Y., Ordas, R., Klein, J.D., and Price, S.R. (2008) Regulation of caspase-3 activity by insulin in skeletal muscle cells involves both PI3-kinase and MEK-1/2. *J. Appl. Physiol.* **105**, 1772–1778.
77. Stitt, T.N., Drujan, D., Clarke, B.A., Panaro, F., Timofeyva, Y., Kline, W.O., Gonzalez, M., Yancopoulos, G.D., and Glass, D.J. (2004) The IGF-1/PI3K/Akt pathway prevents expression of muscle atrophy-induced ubiquitin ligases by inhibiting FOXO transcription factors. *Mol. Cell* **14**, 395–403.
78. Gupte, A.A., Bomhoff, G.L., and Geiger, P.C. (2008) Age-related differences in skeletal muscle insulin signaling: the role of stress kinases and heat shock proteins. *J. Appl. Physiol.* **105**, 839–848.

79. de Alvaro, C., Teruel, T., Hernandez, R., and Lorenzo, M. (2004) Tumor necrosis factor alpha produces insulin resistance in skeletal muscle by activation of inhibitor kappaB kinase in a p38 MAPK-dependent manner. *J. Biol. Chem*. **279**, 17070–17078.
80. Wu, M., Katta, A., Gadde, M.K., Liu, H., Kakarla, S.K., Fannin, J., Paturi, S., Arvapalli, R.K., Rice, K.M., Wang, Y., and Blough, E.R. (2009) Aging-associated dysfunction of Akt/protein kinase B: S-nitrosylation and acetaminophen intervention. *PLoS One* **4**, e6430.
81. Lassus, P., Opitz-Araya, X., and Lazebnik, Y. (2002) Requirement for caspase-2 in stress-induced apoptosis before mitochondrial permeabilization. *Science* **297,** 1352–1354.
82. Ogata, T., Machida, S., Oishi, Y., Higuchi, M., and Muraoka, I. (2009) Differential cell death regulation between adult-unloaded and aged rat soleus muscle. *Mech. Ageing Dev.* **130,** 328–336.
83. Malmgren, L.T., Jones, C.E., and Bookman, L.M. (2001) Muscle fiber and satellite cell apoptosis in the aging human thyroarytenoid muscle: a stereological study with confocal laser scanning microscopy. *Otolaryngol. Head Neck Surg*. **125,** 34–39.
84. Park, S.Y., Kim, H.Y., Lee, J.H., Yoon, K.H., Chang, M.S., and Park, S.K. (2010) The age-dependent induction of apoptosis-inducing factor (AIF) in the human semitendinosus skeletal muscle. *Cell. Mol. Biol. Lett*. **15,** 1–12.
